# Premature Myocardial Infarction in Severe Hypothyroidism: A Case Report

**DOI:** 10.7759/cureus.109722

**Published:** 2026-05-27

**Authors:** Tambi Isaac, Khatun Mstkhaleda, Roxana Lazarescu, Samy Matta

**Affiliations:** 1 Medical Academy, Kabardino-Balkarian State University, Nalchik, RUS; 2 Internal Medicine, Wyckoff Heights Medical Center, New York, USA; 3 Internal Medicine, Alexandria University, Alexandria, EGY

**Keywords:** accelerated atherosclerosis, coronary artery disease, hypothyroidism, hypothyroidism and atherosclerosis, myocardial infarction

## Abstract

Hypothyroidism exerts significant effects on the cardiovascular system through alterations in lipid metabolism, endothelial function, vascular resistance, and myocardial performance. Both overt and subclinical hypothyroidism have been associated with an increased risk of atherosclerosis and premature coronary artery disease, particularly in women and in individuals with markedly elevated thyroid‑stimulating hormone (TSH) levels.

We report the case of a 60‑year‑old woman with long‑standing hypothyroidism, noncompliant with levothyroxine therapy for about six months before presentation. The patient arrived at the emergency department with acute substernal chest pain and was found to have a rapidly rising high‑sensitivity troponin level, which peaked at 125,000, with no ST elevation on electrocardiogram (ECG), consistent with non-ST‑elevation myocardial infarction (NSTEMI). Coronary angiography revealed a critical proximal left anterior descending artery occlusion requiring percutaneous coronary intervention. Echocardiography demonstrated reduced left ventricular ejection fraction (15-20%) and elevated left ventricular end‑diastolic pressure (>16 mmHg), indicating combined systolic and diastolic dysfunction. Thyroid function testing confirmed profound hypothyroidism (TSH >500).

This case illustrates the multifactorial cardiovascular consequences of untreated hypothyroidism and highlights its potential association in precipitating premature ischemic events, even in the absence of traditional cardiovascular risk factors. The patient’s presentation aligns with established pathophysiologic mechanisms linking thyroid hormone deficiency to endothelial dysfunction, dyslipidemia, increased systemic vascular resistance, and myocardial impairment.

This report emphasizes the importance of recognizing hypothyroidism as a modifiable contributor to cardiovascular disease. Routine assessment of thyroid function should be considered in patients presenting with acute coronary syndromes, particularly when traditional risk factors are absent or insufficient to explain the severity of the disease. Early diagnosis and adherence to thyroid hormone replacement therapy may reduce cardiovascular risk and prevent serious complications.

## Introduction

Hypothyroidism is a clinical syndrome characterized by insufficient thyroid hormone production, most commonly due to iodine deficiency, autoimmune thyroiditis (Hashimoto’s disease), or iatrogenic causes following treatment for hyperthyroidism.

The worldwide prevalence of hypothyroidism ranges from 0.3% to 12%, depending heavily on regional iodine intake, with higher rates in iodine-deficient areas and commonest among women and older adults [1]. In the United States, the prevalence of overt hypothyroidism is approximately 0.3% (about 1 in 300 people), while the combined prevalence of subclinical and overt hypothyroidism ranges from 0.3% to 3.7% depending on the definition used and the population studied [1,2].

Common symptoms of hypothyroidism include fatigue, weight gain, constipation, dry skin, cold intolerance, and depressive symptoms. Physical examination may reveal a goiter, facial puffiness, non-pitting pretibial edema, and coarse hair.

Hypothyroidism is approximately six times more prevalent in women, and its incidence increases with age, particularly among individuals older than 60 years [3]. When unrecognized or inadequately treated, thyroid dysfunction can adversely affect metabolic processes and multiple organ systems. The cardiovascular system is among the most significantly impacted, as thyroid hormone receptors are widely expressed in cardiac myocytes and vascular tissues. This receptor distribution explains the broad spectrum of cardiovascular manifestations observed in both hypo- and hyperthyroid states. Notably, even subclinical thyroid dysfunction has been associated with measurable adverse cardiovascular effects [4,5].

Given that our case centers on severe hypothyroidism, we focus here on the proposed mechanisms by which hypothyroidism influences cardiovascular physiology.

## Case presentation

A 60-year-old woman with a long-standing history of hypothyroidism presented to the emergency department with new-onset, severe substernal chest pain that began approximately two hours before arrival. She had been diagnosed with hypothyroidism more than 15 years earlier but had discontinued levothyroxine therapy (home dose: 100 mcg daily) approximately six months before presentation. The patient’s body mass index (BMI) was 34.1 kg/m², and her HbA1c was 5.2% (reference range: 4.0%-5.6%). She reported no family history of premature coronary artery disease, no history of tobacco use, or known hyperlipidemia.

On initial evaluation, she was alert, fully oriented, and hemodynamically stable, with vital signs within normal limits. She denied experiencing typical hypothyroid symptoms such as fatigue, somnolence, or cognitive slowing. Her initial electrocardiogram showed no ST-segment elevation, depression, or T-wave inversions. The first high-sensitivity troponin level was mildly elevated at 98 ng/L; however, given the typical nature of her chest pain and persistence of symptoms, she was admitted for further evaluation. Physical examination was unremarkable except for a nodule on the right thyroid lobe. Thyroid ultrasound showed a very small thyroid gland with a cystic nodule at the superior pole of the right thyroid lobe.

The patient received antiplatelet loading, and a repeat troponin obtained 90 minutes later demonstrated a dramatic rise to 19,693 ng/L (Table 1). In the setting of significant biomarker elevation and ongoing symptoms, she was diagnosed with a high-risk non-ST-elevation myocardial infarction (NSTEMI), and percutaneous coronary intervention (PCI) was scheduled immediately.

**Table 1:** Laboratory values upon presentation. The Siemens Vista high-sensitivity troponin I (TnI-HS) method.

Test	Value	Normal range
Hg	14.7 g/dL	12.0-15.5 g/dL
Ht	42.7%	36%-46%
Na	139 mmol/L	135-145 mmol/L
K	3.9 mmol/L	3.5-5.0 mmol/L
Cr	1.38 mg/dL	0.6-1.1 mg/dL
Cholesterol	217 mg/dL	<200 mg/dL
LDL	165 mg/dL	<100 mg/dL
HDL	44 mg/dL	>40 mg/dL
Triglycerides	75 mg/dL	<150 mg/dL
Troponin 1*	98.5 ng/L	Typically <20 ng/L
Troponin 2*	19693.9 ng/L	Typically <20 ng/L

In preparation for the planned left heart catheterization, which would require the use of iodinated contrast, and given her known history of thyroid hormone nonadherence, thyroid function testing was obtained. Results confirmed profound hypothyroidism consistent with prolonged discontinuation of levothyroxine therapy (Table 2).

**Table 2:** Results of thyroid function testing. TSH, thyroid-stimulating hormone

Test	Value	Normal range
TSH	>500 mIU/L	0.4-4.5 mIU/L
FT3	1.49 ng/dL	2.00-4.40 ng/dL
FT4	0.2 ng/dL	0.8-1.8 ng/dL
Thyroid peroxidase antibodies	420.0 IU/mL	<35 IU/mL
Thyroglobulin antibodies	>4,000	<40 IU/mL

**Figure 1:**
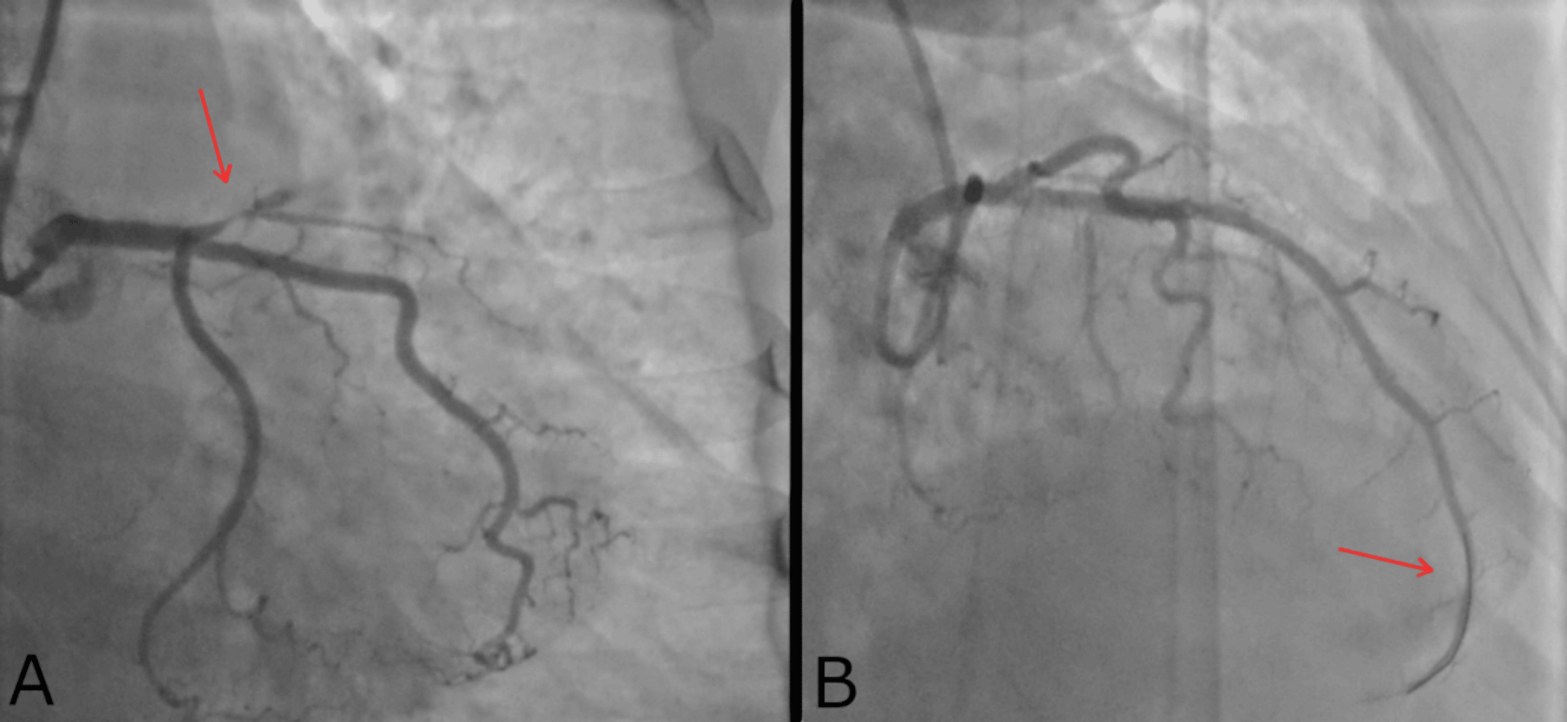
(A) Left coronary angiogram showing 100% occlusion of the left anterior descending artery (LAD) (red arrow); (B) post-stent placement angiogram demonstrating restoration of TIMI III flow in the LAD (red arrow). TIMI, thrombolysis in myocardial infarction

The patient subsequently underwent left heart catheterization, which revealed a critical occlusion of the proximal left anterior descending (LAD) artery (Figure 1A), resembling a thrombotic lesion. She underwent successful PCI with deployment of a drug-eluting stent and percutaneous transluminal coronary angioplasty (PTCA) of the proximal LAD (Figure 1B).

Hemodynamic assessment during the procedure demonstrated elevated left ventricular end-diastolic pressure (LVEDP ~ 37 mmHg), consistent with impaired diastolic relaxation.

**Figure 2:**
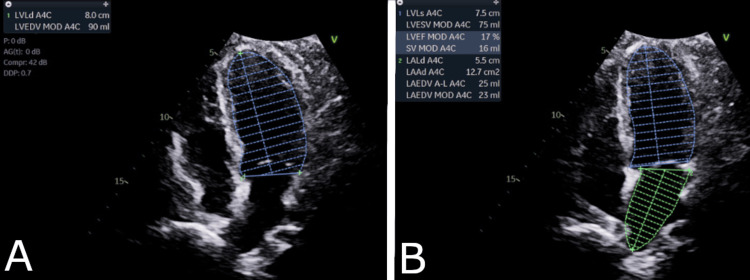
Transthoracic echocardiogram showing (A) end-diastolic frame of the left ventricle and (B) end-systolic frame of the left ventricle.

A transthoracic echocardiogram obtained after the intervention revealed a reduced left ventricular ejection fraction (EF, 15%-20%), indicating systolic dysfunction with global hypokinesis and regional variation, grade I diastolic dysfunction, mild mitral and tricuspid regurgitation, and RVSP ~16 mmHg, which is normal. The combination of depressed systolic function and elevated LVEDP (Figures 2A, 2B) suggested significant myocardial compromise, possibly reflecting the combined impact of severe, untreated hypothyroidism and acute ischemic injury from the LAD occlusion.

The patient recovered well following PCI and was discharged home on hospital day four with dual antiplatelet therapy (DAPT), a high‑intensity statin, guideline-directed medical therapy (GDMT) for heart failure, and levothyroxine 75 mcg daily. Follow‑up appointments were arranged with cardiology, endocrinology, and primary care. During follow-up visits to cardiology and endocrinology, the patient’s thyroid function was reestablished, and a follow-up echocardiogram showed improvement in the EF to 35%.

## Discussion

Thyroid hormones exert broad regulatory effects across multiple organ systems, with the cardiovascular system being among the most profoundly affected.

Both overt and subclinical hypothyroidism have been associated with an increased risk of atherosclerotic cardiovascular disease and myocardial infarction. Several population‑based studies, including the Rotterdam Study, have demonstrated that individuals, particularly women, with subclinical hypothyroidism and thyroid peroxidase antibodies have a higher incidence of coronary artery disease and adverse cardiovascular events [6]. This association is especially pronounced in patients with thyroid‑stimulating hormone (TSH) levels greater than 10 mU/L, and importantly, the elevated cardiovascular risk appears to be reversible with appropriate thyroid hormone replacement therapy [7].

Although hypothyroidism has not been definitively linked to worse outcomes after myocardial infarction, it is associated with an accelerated progression of atherosclerosis and may contribute to premature cardiovascular events [8]. This is particularly relevant in patients without traditional cardiovascular risk factors.

Pathophysiology

Hypothyroidism affects the cardiovascular system through several interrelated mechanisms involving vascular function, myocardial performance, and systemic metabolic regulation.

Vascular Effects and Atherosclerosis

Thyroid hormones are essential for maintaining endothelial integrity and vascular tone, and in hypothyroidism, their deficiency leads to reduced endothelial nitric oxide production with resulting vasoconstriction, increased systemic vascular resistance that often elevates diastolic blood pressure, impaired lipid metabolism that raises LDL cholesterol and triglyceride levels, and additional hemostatic abnormalities and insulin resistance that compound vascular injury; together, these interrelated changes accelerate the development and progression of atherosclerotic plaque [7,9,10].

Effects on Cardiac Function

Thyroid hormones are critical for maintaining normal myocardial contractility and relaxation, and their deficiency disrupts cardiac performance through multiple interconnected pathways. In hypothyroidism, impaired calcium handling and slowed cross‑bridge cycling lead to diastolic dysfunction, with reduced myocardial relaxation evident even at rest, while systolic dysfunction becomes more pronounced during exertion when the heart is unable to adequately increase stroke volume or cardiac output. These abnormalities arise from both genomic mechanisms - such as altered expression of key contractile proteins, β-adrenergic receptors, and calcium-regulating enzymes - and nongenomic mechanisms, including changes in ion channel activity, vascular smooth muscle tone, and peripheral vascular resistance. Together, these effects diminish overall cardiovascular efficiency, reduce exercise tolerance, and heighten susceptibility to myocardial ischemia by limiting coronary blood flow reserve and impairing myocardial work efficiency despite reduced overall oxygen demand [7,10].

Systemic Hemodynamic Consequences

The combination of increased vascular resistance, reduced cardiac efficiency, and accelerated atherosclerosis creates a hemodynamic environment that predisposes patients to ischemic events. Even mild or subclinical hypothyroidism can meaningfully increase cardiovascular risk, particularly in older adults or those with underlying comorbidities.

What Is Relevant About This Case?

This patient lacked conventional cardiovascular risk factors and was younger than the typical age threshold for myocardial infarction in women, which is generally considered premature before 65 years (55 years in men), as previously described in our case series by Matta et al. [11]. Her coronary angiography revealed a proximal LAD occlusion, and concurrent echocardiographic findings of reduced left ventricular ejection fraction and elevated LVEDP indicated both systolic and diastolic dysfunction.

These findings strongly support the hypothesis that chronic, untreated hypothyroidism contributed not only to the development of atherosclerotic disease but also to impaired myocardial performance. The relationship between hypothyroidism and cardiovascular disease has been recognized for decades, dating back to autopsy studies of patients with myxedema that revealed diffuse atherosclerosis [12]. Contemporary evidence continues to show that poorly controlled hypothyroidism increases the risk of coronary artery disease, heart failure, and cerebrovascular events [13].

## Conclusions

This case illustrates the potential cardiovascular consequences of prolonged, untreated hypothyroidism, presenting as premature coronary artery disease in a patient whose traditional risk factors alone may not fully account for the time frame to develop cardiovascular disease or for the severity of the atherosclerotic burden. The well-characterized effects of thyroid hormone deficiency on lipid metabolism, endothelial function, and vascular homeostasis provide a plausible pathophysiological link, though causality cannot be established from a single case. More importantly, this case underscores several actionable clinical takeaways. First, thyroid function testing should be considered in patients presenting with premature or unexplained atherosclerotic disease, consistent with major society recommendations to screen for thyroid dysfunction in the setting of coronary heart disease, heart failure, and dyslipidemia. Second, once hypothyroidism is diagnosed, the importance of medication adherence and adequate hormonal replacement cannot be overstated - persistent subclinical or overt hypothyroidism represents a modifiable cardiovascular risk factor that, if left unaddressed, may accelerate atherogenesis. Third, clinicians should incorporate thyroid status into comprehensive cardiovascular risk assessment and pursue aggressive management of associated metabolic derangements, including dyslipidemia and hypertension, which may be refractory to standard therapies in the absence of euthyroidism.

Ultimately, this case serves as a reminder that early detection, consistent treatment, and close follow-up of hypothyroidism are essential components of cardiovascular risk reduction, and that failure to address thyroid dysfunction may undermine otherwise appropriate preventive strategies.
